# Terahertz wave modulation properties of graphene with different excitation laser power

**DOI:** 10.1039/d2ra04133b

**Published:** 2022-09-26

**Authors:** Shaohang Chen, Ruizhao Yang, Yanni Zhou, Binyi Qin, Yun Li, Jincun Zheng, Yizhi Liang, Tinghui Li, Jianming Liu

**Affiliations:** School of Electronic and Automation, Guilin University of Electronic Technology Guilin 541004 China; Key Laboratory of Complex System Optimization and Big Data Processing, Guangxi Colleges and Universities, Yulin Normal University Yulin 537000 China; School of Electronic and Automation, Guilin University of Aerospace Technology Guilin 541004 China; Research Center of Intelligent Information and Communication Technology, School of Physics and Telecommunication Engineering, Yulin Normal University Yulin China qby207@163.com; School of Chemistry and Food Science, Yulin Normal University Yulin 537000 China; Optoelectronic Information Research Center, School of Physics and Telecommunication Engineering, Yulin Normal University Yulin 537000 China wendy_workemail@163.com; Clooege of Electronic Engineering, Guangxi Normal University Yulin 537000 China

## Abstract

The terahertz wave modulation properties of graphene were investigated using an external 975 nm continuous wave laser with different power. Upon excitation laser, the transmission and modulation depth was measured using terahertz time-domain spectroscopy. The experimental results showed that the modulation depth of monolayer graphene and 3-layer graphene was 16% and 32% under the 1495 mW excitation power. Further, we analyzed the graphene modulation mechanism based on the Drude model and the thin-film approximation. Both theoretical analysis and calculation results showed that the terahertz wave could be modulated using graphene with different excitation laser power.

## Abstract 2

Terahertz wave modulation properties of graphene are investigated using an external 975 nm continuous wave laser. The modulation depth of monolayer graphene and 3-layer graphene was 16% and 32% under 1495 mW excitation power.

## Graphical abstract

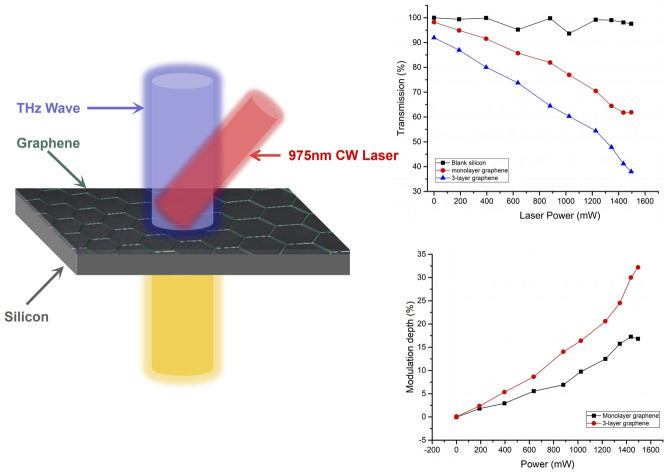

## 1 Introduction

The terahertz (THz) wave is an electromagnetic wave that falls between the infrared and microwave wavelengths. Because of its strong penetration, low energy, spectral fingerprint, coherence, and large communication bandwidth,^1,2^ it has broad application prospects in biomedical diagnosis,^3,4^ high-sensitivity sensing,^5,6^ security inspection,^7^ high-speed communication,^8,9^ nondestructive testing^10–14^ and other fields.^15,16^ Terahertz radiation generation and detection technologies have advanced quickly in recent years. However, the modulation of terahertz wave technology has developed slowly and is restricting the development of terahertz technology. Thus, modifying the terahertz wave is a popular area of study.

Graphene is a typical two-dimensional material that consists of sp^2^ hybridized carbon atoms. Due to its excellent optical, electrical, and magnetic properties, graphene has crucial applications in field-effect transistors, liquid crystal displays, nanocomposites, photodetectors, light-emitting devices, microwave transistors, ultrafast lasers, photovoltaic cells, sensing and energy storage materials.^17,18^ Zhang *et al.*^19^ reported the first graphene light modulator; a graphene layer was placed on top of the optical waveguide in the modulator, and a driving voltage was used to control the graphene layer’s Fermi energy level. To modulate terahertz waves, Sensale-Rodriguez *et al.*^20,21^ designed a transmissive and reflective terahertz modulator. The terahertz wave was modulated by adjusting the gate voltage. The transmissive and reflective terahertz modulators had maximum modulation depths of 15% and 64%, respectively. Wu *et al.*^22^ developed a graphene-based terahertz modulator by filling an ionic gel material between two monolayers of graphene. This structure could enhance the electric field modulation effect on graphene’s Fermi energy level. The modulation depth was 83% when the voltage was 3 V. The modulation methods described above are based on the electrically-driven mode. However, the influence of the charging and discharging process severely restricts the response speed of the electrically controlled terahertz wave. The issue of constrained response speed can be resolved by modulating the terahertz wave in an optically-driven mode. Weis *et al.*^23^ reported using femtosecond laser excitation to achieve a wide band tunability of the THz transmission. They observed a maximum amplitude modulation depth of 99%. However, there was only a maximum 18% difference between the transmission through bare silicon and the sample. Compared with pulsed femtosecond lasers, continuous wave (CW) lasers are more convenient and cost-effective for implementing all-optical terahertz modulators. Wen *et al.*^24^ demonstrated an all-optical terahertz modulator based on monolayer graphene on germanium, which could be driven by a 1.55 μm CW laser. The maximum difference between the transmission through the germanium and the sample was 30%. Fu *et al.*^25^ fabricated a THz modulator consisting of monolayer graphene on a silicon substrate. The experimental results showed that the modulation depth of the transmission could reach 74% for the proposed modulator under an external 450 nm continuous-wave laser photo-excitation. And the difference in transmission between the sample and the silicon substrate can reach a maximum of 49.3%. Du *et al.*^26^ reported an active broadband THz wave modulator based on the graphene-silicon hybrid structure under photoexcitation of a 532 nm CW laser. Previous reports have discussed the optically-driven modulation properties of monolayer graphene. However, there are rare reports about optically-driven multilayer graphene.

In this paper, we analyzed the THz modulation properties of monolayer and 3-layer graphene. We used a 975 nm CW laser as the excitation source. The penetration depth of the silicon substrate is different under different exciting wavelengths. The excitation source used in this study had a larger penetration depth in the silicon substrate than in the previous reports. With 975 nm CW laser illumination, the transmission and modulation depth was measured using terahertz time-domain spectroscopy (THz-TDS) under different excitation power. We observed significant transmission attenuation and increased modulation depth when the excitation laser power was tuned. And then, we revealed the graphene modulation mechanism using the Drude model and the thin-film approximation.

## 2 Materials and method

### 2.1 Experimental system

**Fig. 1:**
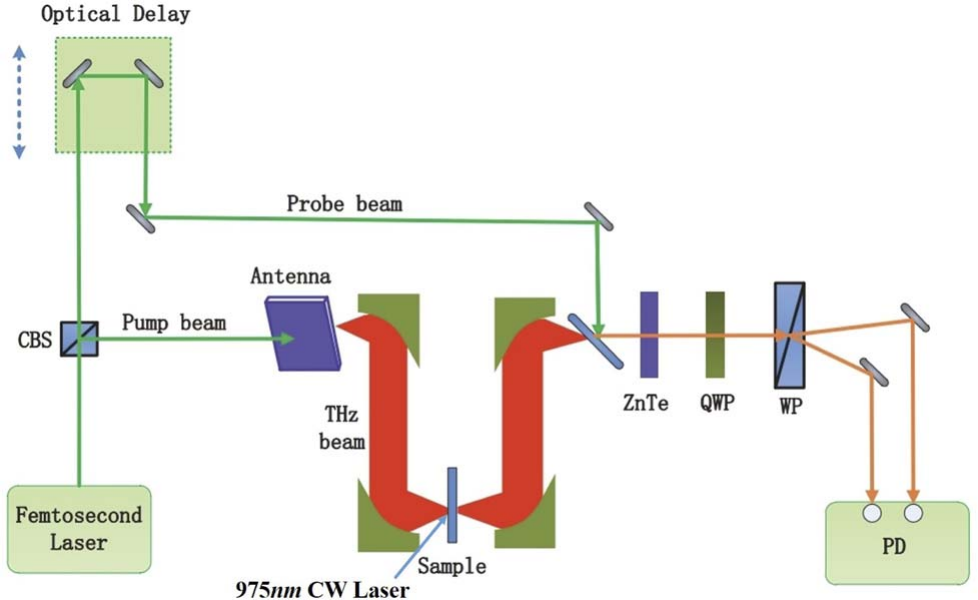
The experimental terahertz time-domain spectroscopy system.

The experimental system contained a THz-TDS and an external 975 nm CW laser as shown in Fig. 1.

The ultrafast femtosecond laser emitted a central wavelength of 780 nm pulse, of which the pulse width was less than 100 fs. Using a beam splitter (CBS), the pulse laser was divided into two paths with different energy. The laser with higher energy served as the pump beam, irradiating the GaAs photoconductive antenna and generating a terahertz beam. The generated terahertz beam was focused on a test sample through the parabolic mirror. Due to the interaction between the test sample and the terahertz beam, the terahertz wave penetrated the sample and carried the sample information. And then, the terahertz beam carrying information from the sample struck the ZnTe electro-optic crystal. On the other hand, the laser with lower energy was used as the probe beam, which met the terahertz beam on the ZnTe electro-optic crystal after the lens collimation and focus. Throughout the ZnTe electro-optic crystal, the probe beam containing the terahertz information of the sample was changed from isotropic polarization to anisotropic polarization. And then, the probe beam was divided into two perpendicular polarized beams using a Wollaston prism. A differential photodetector (PD) collected the two perpendicular polarized beams. At last, the terahertz time-domain spectrum was obtained by the PD collected signal. Moreover, a 975 nm CW laser was selected as an excitation laser.

### 2.2 Time-domain spectral data acquisition

The monolayer and 3-layer graphene samples were purchased from Shenzhen Six Carbon Technology Co., Ltd. We measured the spectral transmission of the graphene samples by standard THz-TDS. We used a 975 nm CW laser for photo-doping the graphene samples. The laser beam was obliquely incident on the sample (from graphene to the silicon substrate) with an angle of 45°, as shown in Fig. 1. We used 10 energy gradients of excitation power (0 mW, 190 mW, 395 mW, 636 mW, 880 mW, 1024 mW, 1226 mW, 1345 mW, 1436 mW, 1495 mW). A time-domain spectrum of the sample was obtained by averaging ten measurements of each sample to eliminate random errors.

## 3 Results and discussion

### 3.1 Time-domain spectral analysis

**Fig. 2:**
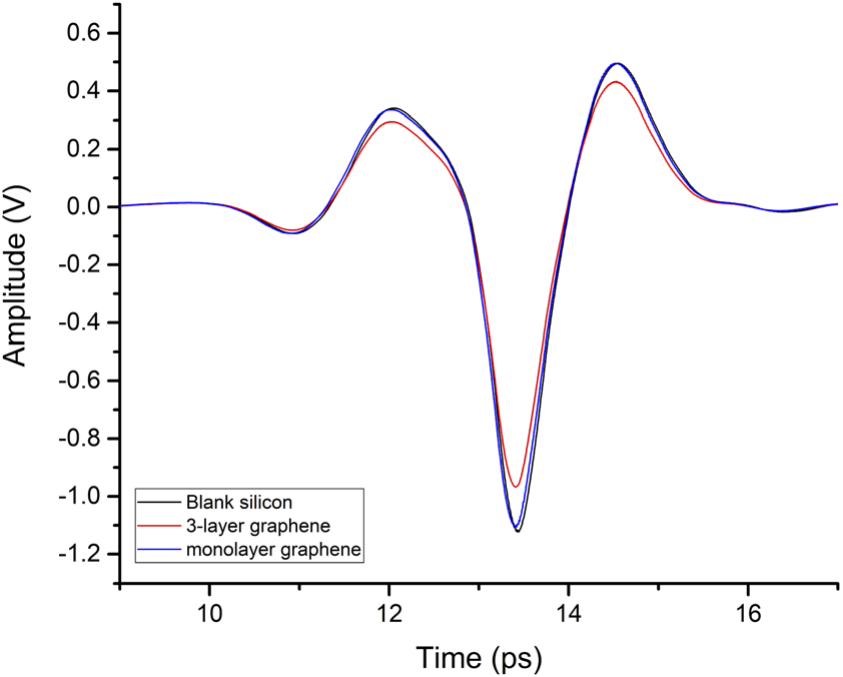
Terahertz time-domain spectra of silicon, monolayer graphene, and 3-layer graphene.

The time-domain spectra of silicon, monolayer graphene, and 3-layer graphene, without external photoexcitation, are shown in Fig. 2.

Taking silicon as a reference, the terahertz amplitude decreased after it penetrated the graphene. In particular, at two peaks (12 ps and 14.5 ps) and one trough (13.4 ps), the terahertz signal decreased to 86.2%, 86.5%, and 86.2% of the reference signal amplitude after passing through 3-layers graphene, respectively. This indicates that graphene absorption of the terahertz signal is subsequently enhanced with increasing layers.

**Fig. 3:**
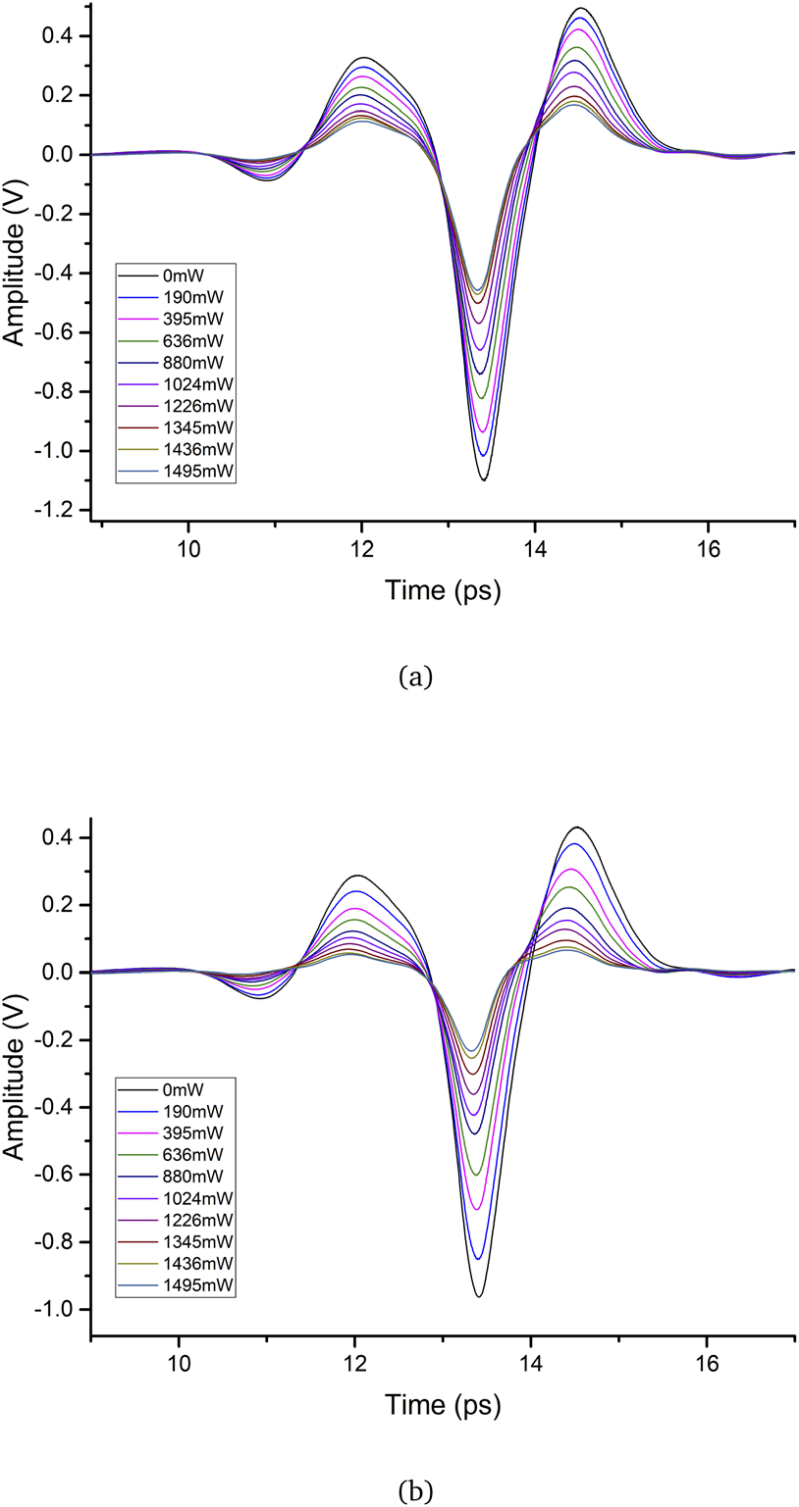
Time-domain spectra of (a) monolayer graphene and (b) 3-layer graphene.

The THz response monolayer graphene and 3-layer graphene under an external excitation of a 975 nm CW laser irradiation is shown in Fig. 3. The excitation laser power was varied from 0 mW to 1495 mW by adjusting the CW laser. When the excitation power was increased from 190 mW to 1495 mW, the time-domain signal amplitude for monolayer and 3-layer graphene significantly decreased. In Fig. 3(a), when the excitation power reached 1495 mW, two peaks (12 ps and 14.5 ps) and one trough (13.4 ps) decreased by 65.2%, 66.1%, and 58.5%, respectively, compared to the monolayer graphene without the excitation laser. As the excitation power increased to 1495 mW in Fig. 3(b), two peaks (12 ps and 14.5 ps) and one trough (13.4 ps) decreased by 81.7%, 84.9% and 76% in comparison to 3-layer graphene without the excitation laser. This demonstrates that under the same excitation power, the 3-layer graphene absorbed the terahertz wave more strongly.

### 3.2 Frequency-domain spectral analysis

The frequency-domain spectra were obtained using a fast Fourier transform of the time-domain signals to quantify the transmission of terahertz waves at different frequencies. The transmission was defined as in eqn (1).1
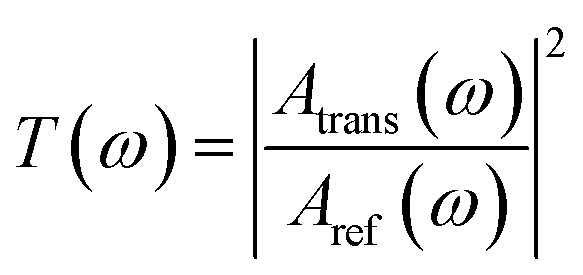
where *A*_trans_(*ω*), *A*_ref_(*ω*) are the amplitude values of the sample and the reference (silicon) after Fourier transform, respectively.

**Fig. 4:**
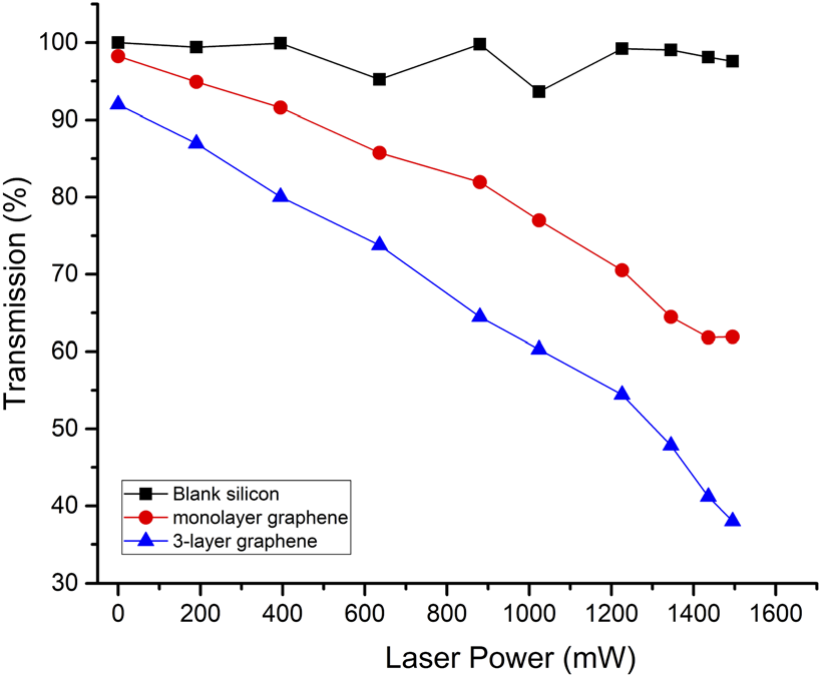
The dependence of the average transmission on the excitation laser power.

The dependence of the average transmission on the excitation laser power is shown in Fig. 4. Comparing graphene with silicon, we observed 2% and 8% transmission decrease for monolayer and 3-layer graphene, respectively, without photo-doping due to interband transitions in graphene.^27,28^ For silicon, the average transmission decreased from 100% without optical excitation to 96% with a pump power of 1495 mW. Upon photo-doping of graphene, the transmission of the THz wave decreased. With a pump power of 1495 mW, the average transmission of monolayer graphene decreased to 62%, and the average transmission of 3-layer graphene decreased to 38%. Compared with the previous reports, we obtained a higher maximum value difference between the transmission through the silicon and graphene using a 975 nm CW laser pump. The maximum difference between the transmission through the silicon and the monolayer graphene or 3-layer graphene, was 34% and 58%, respectively. This was because the more photo-generated carriers could be excited using a longer wavelength laser. Furthermore, the THz beam attenuation was significantly enhanced compared to silicon at equivalent power levels of the photo-doping graphene. This showed that graphene was dominant in the THz wave modulation compared with silicon.

Furthermore, the modulation depth was defined as follows.2
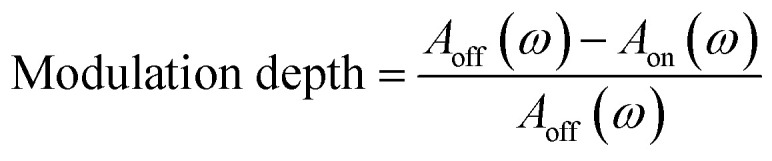
where *A*_off_(*ω*) and *A*_on_(*ω*) denote the terahertz amplitudes without and with excitation laser, respectively.

**Fig. 5:**
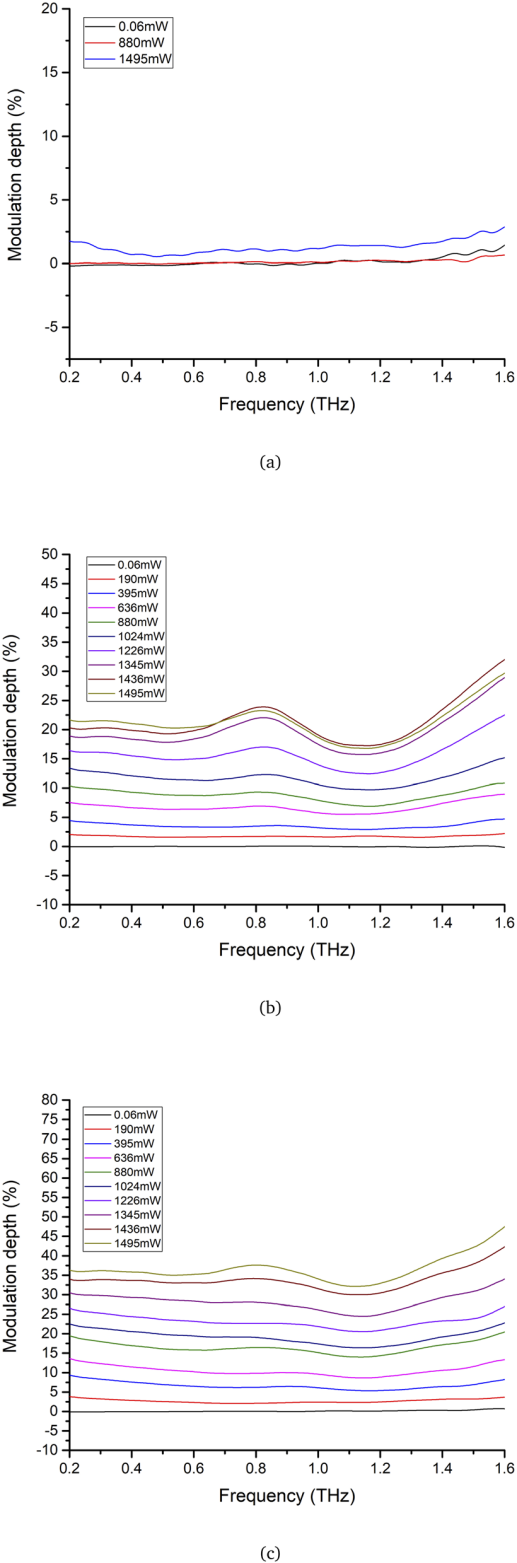
The modulation depth of (a) silicon, (b) monolayer graphene and (c) 3-layer graphene.

Fig. 5 displays the modulation depth of silicon, monolayer graphene, and 3-layer graphene with various excitation powers in the 0.2 THz to 1.6 THz range. The modulation depth of silicon, monolayer graphene, and 3-layer graphene was about 0.6%, 16%, and 32%, respectively, when the excitation power was 1495 mW. The 3-layer graphene has a deeper modulation depth than monolayer graphene and silicon at the same excitation power.

**Fig. 6:**
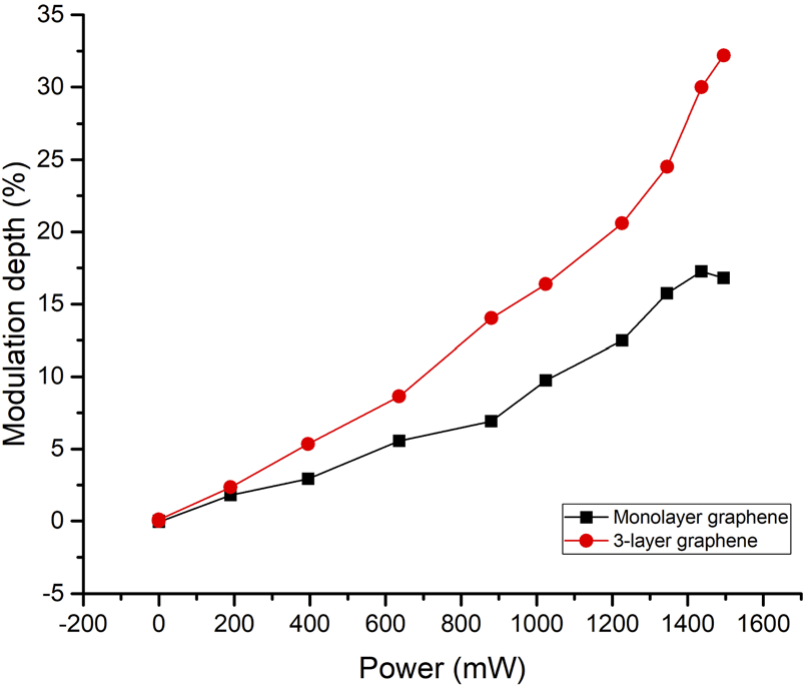
The monolayer and 3-layer graphene modulation depth at 1.14 THz under different excitation power.

The modulation depth of monolayer and 3-layer graphene at 1.14 THz is depicted in Fig. 6. With an increase in excitation power, the modulation depth increased. The modulation depth of 3-layer graphene and monolayer graphene were comparable in the 0 mW to 200 mW range. The modulation depth of 3-layer graphene eventually grew bigger than that of monolayer graphene with increased excitation power. This indicates that the modulation performance of 3-layer graphene is better than monolayer graphene.

### 3.3 Analysis of the graphene modulation mechanism

When the excitation laser was incident from the graphene side, a small portion of the excitation laser was absorbed by graphene. It caused photo-generated free carriers to arise from the graphene. The nonabsorbed transmitted portion of the excitation laser penetrated the silicon substrate. Most of the energy was completely absorbed within the penetration length defined by the absorption coefficient. Because the amount of absorbed energy of silicon was larger than in graphene, the major fraction of photo-generated free carriers were generated in silicon. The illuminated area and the penetration length of the excitation laser in silicon defined the free carriers within a volume. And then, the photo-generated free carriers diffused into graphene and accumulated at the interface of the silicon substrate and graphene until a steady state was attained. These free carriers arriving in graphene experienced much higher mobility than their silicon counterparts. This resulted in a more significant change of conductivity than in silicon. The free carriers induced a change in the electric conductivity, which was inherently correlated with decreased THz transmission through the material. Thus, the THz transmission and modulation depth depended on the conductivity change.

The monolayer graphene conductivity in the terahertz range complied with the Drude model, as follows.^29^3
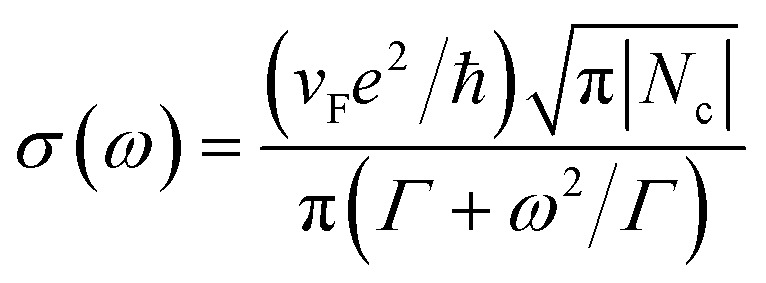
where *e*, ℏ, *ω*, *v*_F_, *Γ*, and *N*_c_ were the elementary charge, Planck’s constant, angular frequency, Fermi velocity, carrier scattering probability and carrier concentration, respectively. Moreover, randomly stacked multilayer graphene can be treated as multiply electronically decoupled monolayer graphene.^30,31^ The silicon prohibited bandwidth was 1.12 eV, while the silicon cutoff wavelength for its intrinsic leap was 1107 nm. We used a 975 nm CW laser as an excitation source to generate photo-generated carriers. The free carriers induced a change in the electric conductivity. Different laser power could excite different amounts of photo-generated charge carriers. Thus, graphene could modulate the terahertz wave with different excitation laser power.

Because the thickness of the graphene was far smaller than the center wavelength of the THz wave, we applied the thin-film approximation^32^ to describe the experimental data. The frequency-dependent equivalent conductivity of graphene could be extracted from the measured transmission spectra using the following formula,4
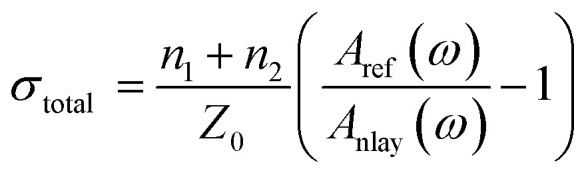
where *n*_1_ is the refractive index of air, *n*_2_ is the refractive index of the graphene substrate, *Z*_0_ = 377 Ω is the vacuum impedance, and *A*_nlay_(*ω*), *A*_ref_(*ω*) are the amplitude values of the sample and the reference after Fourier transform.

**Fig. 7:**
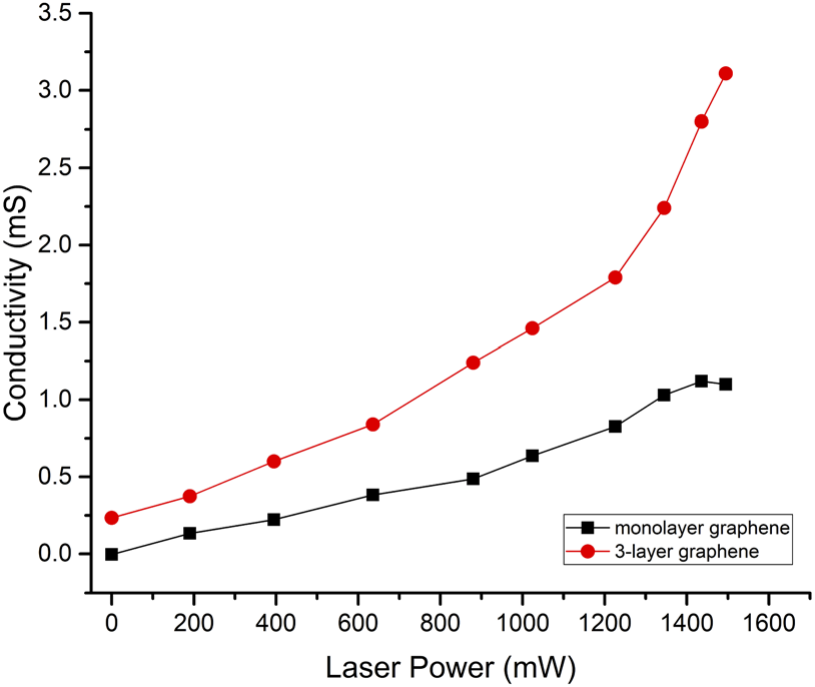
The relation between excitation power and graphene conductivity.

The relation between excitation power and graphene conductivity is shown in Fig. 7. It is evident that the graphene conductivity increases nonlinearly with pump power. When the pump power was 0 mW, the monolayer graphene conductivity was close to zero. As the excitation power increased, more photo-generated carriers were excited. This led to monolayer graphene conductivity increase. As the pump power rose to 1495 mW, the monolayer graphene conductivity increased to 1.1 mS. The 3-layer graphene conductivity mirrors this behavior: the 3-layer graphene conductivity was 0.23 mS as the pump power was 0 mW, and when the pump power increased to 1495 mW, the conductivity was 3.11 mS.

## 4 Conclusions

In this paper, we investigated the terahertz wave modulation properties of graphene with different excitation CW laser power. Under excitation from a 975 nm CW laser in 10 gradients, the monolayer and 3-layer graphene terahertz transmission and modulation depth were measured using the THz-TDS system. Different from previous reports, we use a 975 nm CW laser as an excitation source. It had a larger penetration depth in the silicon substrate than in previous reports, which excited more photo-generated carriers. Thus, a higher maximum value difference between the transmission through the silicon and graphene than in previous reports was obtained. For the graphene, the THz transmission decreased with increasing layer number or excitation power. When the excitation power was 1495 mW, the modulation depth of monolayer graphene and 3-layer graphene was above 16% and 32%, respectively. The Drude model and standard thin-film approximation were employed to analyze the graphene modulation mechanism. The variation of graphene conductivity increased with increasing laser power, which influenced the modulation depth. Therefore, the terahertz wave could be modulated using graphene with different excitation laser power. Optically tuned graphene is a potential method for realizing a terahertz wave active amplitude modulator in future applications.

## Author contributions

Conceptualization – Shaohang Chen, Binyi Qin and Jianming Liu; data curation – Binyi Qin; formal analysis – Shaohang Chen, Binyi Qin, Yizhi Liang and Jincun Zheng; funding acquisition – Binyi Qin, Ruizhao Yang, Tinghui Li and Yanni Zhou; investigation – Ruizhao Yang, Tinghui Li and Yun Li; methodology – Shaohang Chen, Binyi Qin, Yanni Zhou and Jincun Zheng; project administration – Binyi Qin; resources – Binyi Qin, Ruizhao Yang and Tinghui Li; software – Yizhi Liang and Jianming Liu; supervision – Binyi Qin and Jianming Liu; validation – Yun Li, Tinghui Li and Yanni Zhou; writing original draft – Shaohang Chen, Ruizhao Yang and Yun Li; writing review and editing – Binyi Qin, Yizhi Liang and Jianming Liu. All authors have read and agreed to the published version of the manuscript.

## Conflicts of interest

There are no conflicts to declare.

## Supplementary Material
